# Robust Non-Contact Monitoring of Respiratory Rate using a Depth Camera

**DOI:** 10.1007/s10877-023-01003-7

**Published:** 2023-04-03

**Authors:** Paul S Addison, André Antunes, Dean Montgomery, Philip Smit, Ulf R. Borg

**Affiliations:** 1 Medtronic Patient Monitoring, Technopole Centre, Edinburgh, UK; 2 Medtronic Patient Monitoring, Boulder, CO USA

**Keywords:** Respiratory rate, Non-contact monitoring, Depth-sensing camera.

## Abstract

**Purpose:**

Respiratory rate (RR) is one of the most common vital signs with numerous clinical uses. It is an important indicator of acute illness and a significant change in RR is often an early indication of a potentially serious complication or clinical event such as respiratory tract infection, respiratory failure and cardiac arrest. Early identification of changes in RR allows for prompt intervention, whereas failing to detect a change may result in poor patient outcomes. Here, we report on the performance of a depth-sensing camera system for the continuous non-contact ‘touchless’ monitoring of Respiratory Rate.

**Methods:**

Seven healthy subjects undertook a range of breathing rates from 4 to 40 breaths-per-minute (breaths/min). These were set rates of 4, 5, 6, 8, 10, 15, 20, 25, 30, 35 and 40 breaths/min. In total, 553 separate respiratory rate recordings were captured across a range of conditions including body posture, position within the bed, lighting levels and bed coverings. Depth information was acquired from the scene using an Intel D415 RealSense^TM^ camera. This data was processed in real-time to extract depth changes within the subject’s torso region corresponding to respiratory activity. A respiratory rate RR_depth_ was calculated using our latest algorithm and output once-per-second from the device and compared to a reference.

**Results:**

An overall RMSD accuracy of 0.69 breaths/min with a corresponding bias of -0.034 was achieved across the target RR range of 4–40 breaths/min. Bland-Altman analysis revealed limits of agreement of -1.42 to 1.36 breaths/min. Three separate sub-ranges of low, normal and high rates, corresponding to < 12, 12–20, > 20 breaths/min, were also examined separately and each found to demonstrate RMSD accuracies of less than one breath-per-minute.

**Conclusions:**

We have demonstrated high accuracy in performance for respiratory rate based on a depth camera system. We have shown the ability to perform well at both high and low rates which are clinically important.

## Introduction

One of the most ubiquitous vital signs measured in the clinical setting is respiratory rate (RR). A significant change in RR is often an early indication of a major complication such as respiratory tract infections, respiratory depression associated with opioid consumption, anesthesia and/or sedation, as well as respiratory failure [1–3]. In addition, many early warning scores (EWS), for example MEWS, NEWS, etc., incorporate respiratory rate within the scoring system [4].

**Fig. 1:**
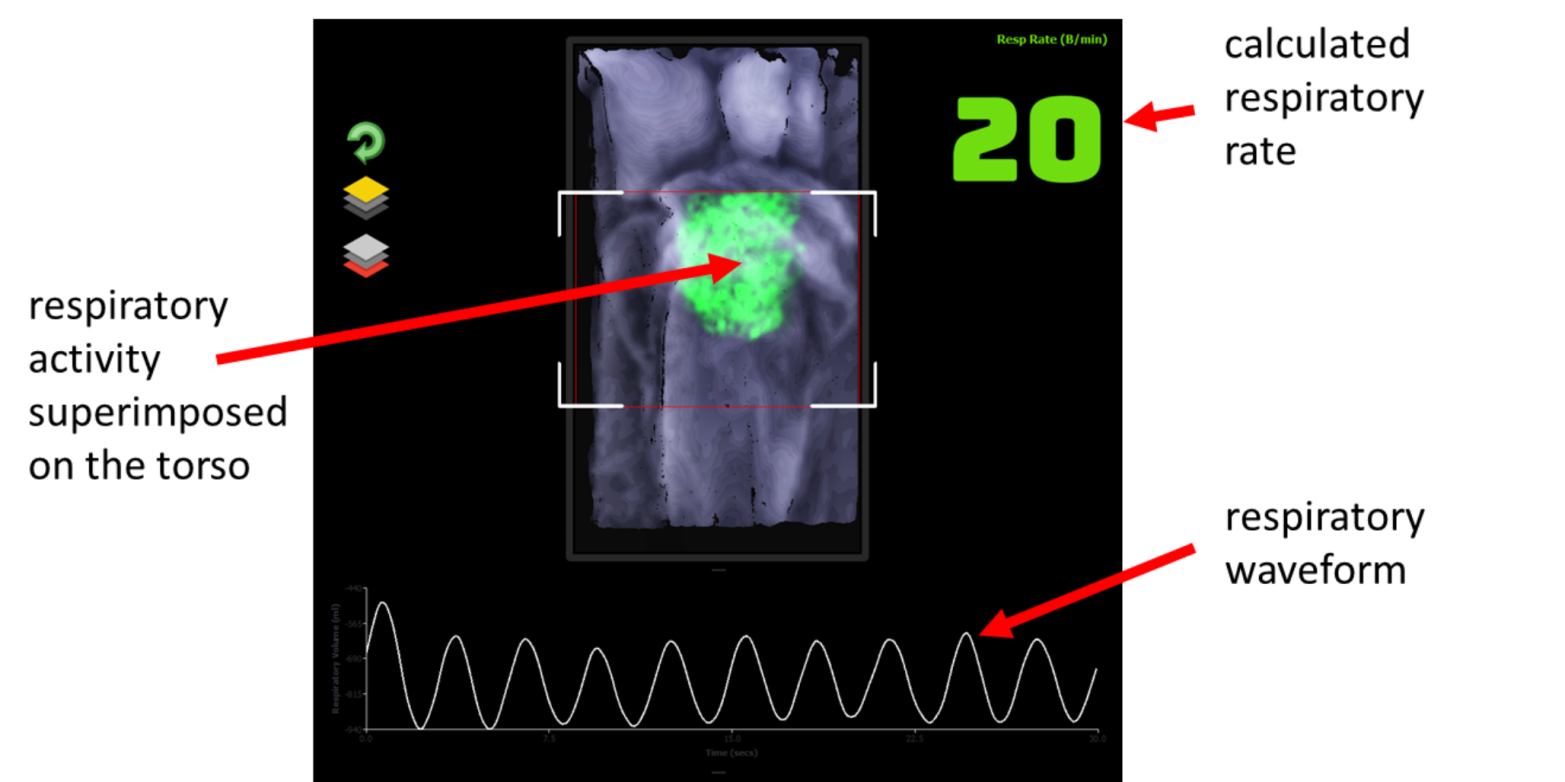
Respiratory Visualization and Waveform using the Depth Camera

Depth cameras are emerging as a tool that can provide a continuous measure of respiratory rate without the need to attach a sensor to the patient. In addition, non-contact monitoring of RR could prove invaluable for infection control during viral pandemics, including influenza and coronavirus, as well as those with other viral respiratory tract diseases, where minimum contact with the patient is desired and a robust measurement is essential [5, 6]. To obtain a respiratory rate using a depth camera, a respiratory signal must first be derived from the respiratory motion of the patient. Figure 1 contains a rendered depth image of a subject lying in a hospital bed, where the region of respiratory activity is highlighted by a color patch superimposed back onto the image. This respiratory activity manifests itself as a change in distances from the camera to the surface of the patient’s torso over time, where the changes cycle with breath period. The frame-to-frame differences in depth may be integrated over a region of interest in the field of view and used to derive a time-varying signal commensurate with respiratory activity. This results in the respiratory signal shown below the depth image in Fig. 1. We have found this respiratory signal to be relatively robust to noise, posture, bedclothes, sheets, etc. Once the respiratory signal has been derived, RR may be calculated from respiratory modulations identified within the signal. A detailed account of non-contact respiratory monitoring using depth sensing cameras is provided in Addison et al., (2021) [7].

Several studies have investigated the derivation of respiratory rate from depth cameras. A study of ICU patients by Her et al. [8] considered the use of depth sensing to measure respiratory rate. They studied 44 ICU patients: 32 under invasive mechanical ventilation and 12 spontaneously breathing under high flow oxygen delivered via a nasal canula. All patients were supine in bed with a 30° angulation of the mattress. They found a bias (mean difference) of less than 1 breaths/min and standard deviation of less than 5 breaths/min. This corresponded to a correlation coefficient of R = 0.91 between the depth measured RR and the reference. A study by Martinez & Stiefelhagen [9] assessed 67 healthy patients in a sleep lab where various objects and coverings were considered including blankets, pillows, books, newspapers and magazines, etc. They found that they could determine RR within 1 breaths/min 88.7% of the time. Yu et al. [10] also investigated RR monitoring during sleep and monitored a subject over 10 nights with and without a blanket. They achieved a 92% accuracy in their measurements for the 42 h of data collected. Seppanen et al. [11] reported small absolute errors between 0.26% and 0.30% when comparing their measurement of RR_depth_ to that of a spirometer in eight volunteers who were instructed to follow a variety of breathing patterns while being monitored. They used regions of interest (ROIs) which mimicked chest/abdomen bands similar to those used in sleep studies. Bernacchia et al. [12] considered breath period in a study of 10 healthy young adults, asking subjects to maintain ‘regular respiratory activity’. A performance corresponding to 9.7% difference in the residuals was obtained between the depth-based RR and a spirometer reference. And Centonze et al. [13] found an average error in frequency of 0.87% when compared to a polysomnographic record when they studied a single patient over 8 h using a Kinect depth sensing camera. Many studies reported to date have covered a limited range of rates and/or conditions. Furthermore, they often using non-regression type statistics, which makes it difficult to compare it to regression statistics which is the norm when comparing a prototype test device to a reference device.

In previous work by our group we took RR measurements of 14 healthy subjects during a hypoxia study used for oximetry device development [14]. A wide range of respiratory rates were exhibited by each participant due to the hypoxic challenge (who were taken down to oxygen saturation levels of 70%). The bias and RMSD accuracy between depth-based respiratory rate (RR_depth_) and the reference rate from a capnograph (RR_cap_) was found to be 0.04 and 0.66 breaths/min respectively. The least squares fit regression equation was determined to be: RR_depth_ = 0.99 × RR_cap_ + 0.13 and the resulting Pearson correlation coefficient, R, was 0.99 (p < 0.001). These results were achieved with a 100% reporting uptime. In another study by our group where we compared RR from a depth camera system and a ventilator, we found bias and RMSD accuracy between RR_depth_ and a ventilator reference, RR_vent_, to be -0.02 breaths/min and 0.51 breaths/min respectively for a series of runs at various ventilator settings [15].

The normal respiratory rate range for adults is generally accepted to be from 12 to 20 breaths/min (with some small variation in this range depending on the information source). Flenady et al. [16] reported that small variations either side of this range of just 4 breaths/min can indicate early signs of serious clinical deterioration that would otherwise go undetected. Thus, accuracy at the high and low end is particularly important to the clinician. It is a relatively simple task to derive a respiratory rate for subjects breathing within the normal range of respiration. The task become considerably more challenging at higher and lower rates: which are the rates of most clinical significance. At high rates, the tidal volumes become commensurately smaller and can result in very small modulations in the depth-based respiratory signal, and these may be subject to high and low frequency noise making the extraction of the actual breathing signal component difficult. At low rates, the respiratory modulations may be of widely varying morphology with extra small peaks, long pauses and vastly different I:E (inhalation:exhalation) ratios. This also makes parsing out individual breaths from other signal components difficult for low rates.

Here, we report on the performance of a depth-sensing camera system based on the Intel RealSense™ D415 depth camera (Intel Corp, Santa Clara, CA) for the continuous non-contact ‘touchless’ monitoring of Respiratory Rate (RR), where we consider various respiratory rates, postures, coverings and lighting conditions. We aimed to develop a system which would cover an operating range from 4 to 40 breaths/min while exhibiting high accuracy at the low and high rates.

## Methods

### Data acquisition and processing

Seven healthy volunteers took part in the study where data was collected over a series of runs where each volunteer undertook a range of breathing rates from 4 to 40 breaths/min. These were set rates of 4, 5, 6, 8, 10, 15, 20, 25, 30, 35 and 40 breaths/min. A total of 553 separate respiratory rates segments were captured across a range of conditions including body posture, position within the bed, lighting conditions and bed coverings.

Depth information was acquired from the scene using an Intel RealSense™ D415 camera connected to a laptop at a frame rate of 15 fps. The camera was mounted on a tripod and placed at approximately 1.1 m above the torso of the subject. A targeting box was included on the display to make it easier to position the camera above the subject. This is indicated by the four white-line box corners that can be seen in Fig. 1. By positioning the camera so that the subject’s torso is within this region, we found that we could enable faster positioning of the camera and ensure good quality data collection throughout the study. The study was conducted during the COVID pandemic and each subject conducted the captures in their own home on a range of mattresses, bed coverings, pillows, indoor lighting conditions, etc. During the test runs, each subject was instructed to follow a screen-based metronome where inhalation and exhalation were dictated by a colored bar filling and emptying across the screen.

This acquired depth data was processed in real-time to extract depth-changes within the region of interest corresponding to the subject’s torso region as described in the previous section and shown in Fig. 1. Respiratory rate, RR_depth_, was calculated using our latest algorithm and output once-per-second from the device. The RR data was then saved on the device for post processing and performance analysis. Two reference signals were used over the testing period depending on availability of device: a capnograph reference (*Capnostream 35*, Medtronic, Boulder, CO) or a spirometer reference (SpiroFlo, *SpiroSonic*, Uscom Kft, Budapest).

**Fig. 2:**
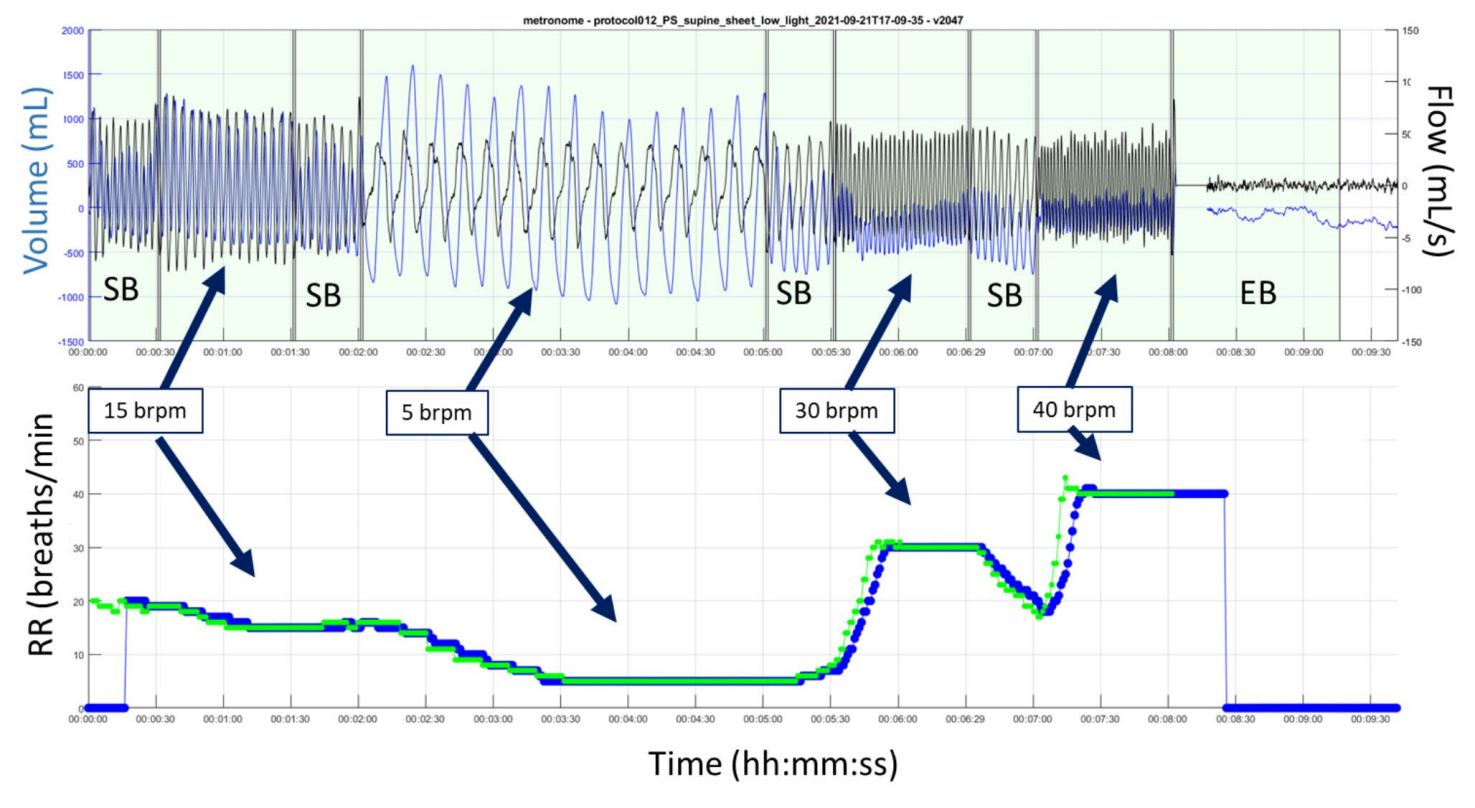
**Example of a Respiratory Signal for a Test where the Subject Breathed at Four Set Rates interspersed with Spontaneous Breathing** Top Plot: The flow (black trace) and volume signal (blue trace) from the depth sensing system Bottom Plot: The computed RR_depth_ (blue) and the corresponding reference RR (green)

In order to avoid overburdening the volunteers, the rates were split into separate sub-protocols where a few rates were conducted at a time. This allowed for the subject to rest adequately between each sub-protocol. In this way we built up a comprehensive data set of respiratory rates from 4 to 40 breaths/min for each subject. Figure 2 shows an example test run segment of data where the subject breathed at the four set rates of 15, 5, 30 and 40 breaths/min, i.e. corresponding to four segments of the 553 captured RR segments. (Other sub-protocols included other rates i.e. 4, 6, 8, 10, 20, 25 and 35 breaths/min.) The top plot contains the respiratory volume signal (in blue) and its derivative (the respiratory flow signal, in black). Both signals are used in our RR algorithm which performs a series of validation checks on the flow signal to determine whether signal features are, in fact, associated with breathing. RR_depth_ was then output once-per-second for comparison with the reference RR. A total of 16,425 separate samples of RR were collected over the 553 separate rate segments.

The lower plot contains the computed RR_depth_ signal plotted with the RR_ref_ from the reference signal (which here is from a capnograph). Between each set rate, the subject was allowed to breathe spontaneously at whatever rate was comfortable. These regions are denoted SB in the plot. At the end of the protocol the subject left the bed. This empty bed region is denoted EB in the plot. Note that the signal segments at the lower rates are longer as fewer breaths are present for a given period. Lengthening all set rate periods was not an option as it is challenging for the subject to breathe at the higher rates for long periods, hence we switched to longer windows only for the lower rates. It can be observed in Fig. 2 that the amplitude of the flow signal diminishes greatly at the higher rates, commensurate with the lower tidal volumes required by the subject to maintain a roughly constant minute volume to avoid hyper- or hypoventilation. These lower signal amplitudes cause the identification of breaths to become significantly more challenging as the breathing signal drops into noise (as described in the previous section).

### Data analysis

*Bias* and *accuracy* statistics were calculated to compare the depth data-derived respiratory rate, RR_depth_, with the reference RR_ref_, where the rates were sampled once per second from the devices and the analysis performed once the rates had settled down to a steady state. These are, respectively, the mean difference (bias) and the root mean squared difference (RMSD) between the test and reference values. That is (using RR as an example):

1 $$bias=\frac{\sum _{i=1}^{N}\left({RR}_{depth}\left(i\right)-{RR}_{ref}\left(i\right)\right)}{N}$$

and.

2 $$RMSD_{accuracy}=\sqrt{\frac{\sum _{i=1}^{N}{\left({RR}_{depth}\left(i\right)-R{R}_{ref}\left(i\right)\right)}^{2}}{N}}$$

where “i” corresponds to the index of each of the N = 16,425 RR samples analysed.

The RMSD represents a combination of the systematic and random components of the differences between the corresponding readings from the two devices. Least-squares linear regression was performed to obtain the line of best fit between the depth and reference parameters from which the gradient, intercept, and Pearson correlation coefficient, R, were computed. A Bland–Altman analysis of the data was also performed using the method of Bland and Altman [17]. The corresponding limits of agreement were calculated using this methodology.

A reliability measure in the form of an uptime was computed. This is a measure of the percentage of time that an RR_depth_ can be successfully computed for each subject. A high uptime is usually a fundamental technical requirement for the development of a medical device. To be acceptable for use in clinical practice, both accuracy and uptime must be sufficiently high. Note that accuracy may be improved at the expense of uptime by avoiding posting results when the signal quality is poor (e.g., due to noise). Hence it is our belief that results for continuous monitoring technologies must provide performance metrics which include uptime. We define uptime, here as the duration, T_valid_, that a valid RR can be calculated and reported by the algorithm as a percentage of the monitoring period, T_mon_, i.e.:

3 $$uptime=\frac{{T}_{vailid}}{{T}_{mon}}$$

Matlab™ (R2022b) was used to perform the statistical analysis. An in-house C + + application was used to capture the depth data and calculate the RR.

## Results

**Fig. 3:**
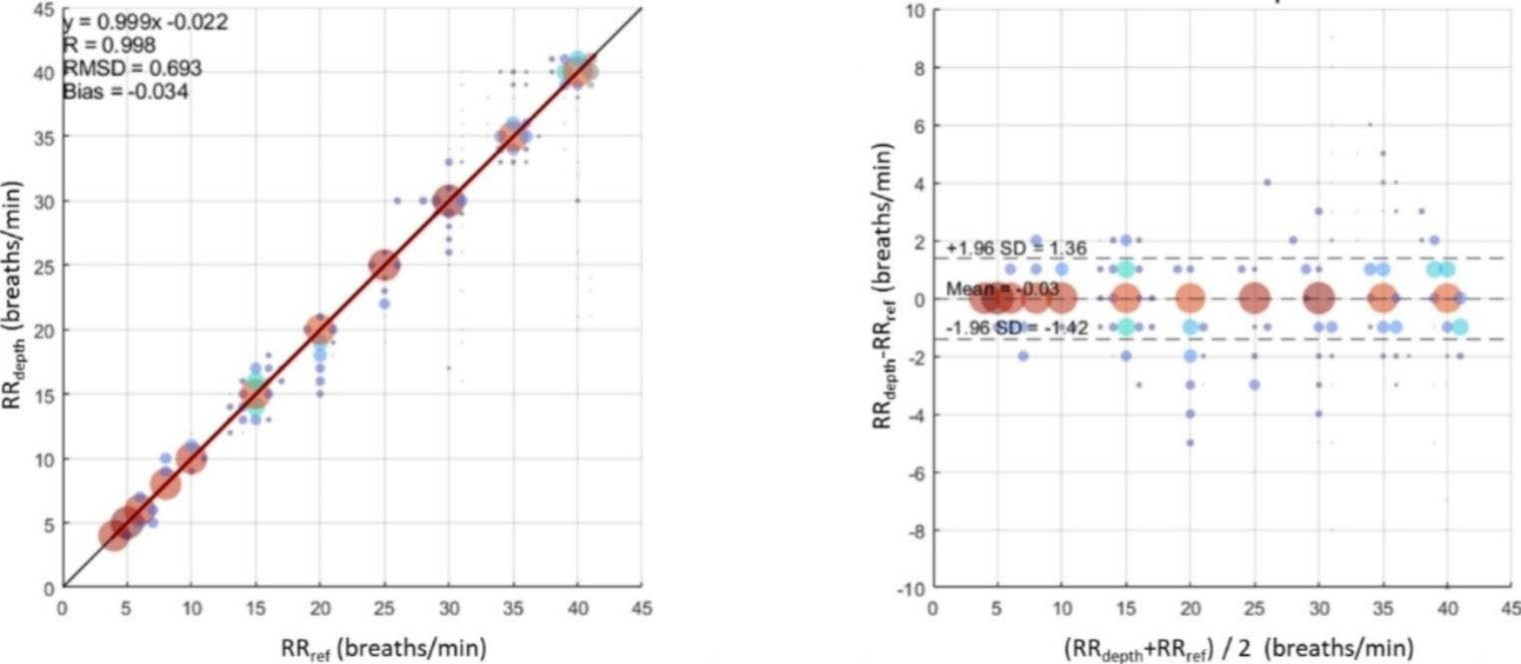
Scatter Plot and corresponding Bland-Altman Plot of Results

Figure 3a contains the scatter plot of RR_depth_ against RR_ref_ pooled together for all experimental runs. A Pearson correlation coefficient of 0.998 was achieved and a line of best fit given by RR_depth_ = 0.999 x RR_ref_ – 0.022 breaths/min. The overall RMSD across the runs was 0.69 breaths/min with a corresponding bias of -0.034 breaths/min respectively. The associated Bland-Altman plot, illustrated in Fig. 3, shows a mean of -0.034 breaths/min and limits of agreement of -1.42 and 1.36 breaths/min. We further broke down the performance of the RR algorithm according to our definition of low, medium and high rates of < 12, 12–20 and > 20 breaths/min. The results are contained in Table 1 where it can be seen that all three respiratory rate ranges - low, normal and high – exhibit RMSD accuracies less than one breath per minute. These results correspond to an overall uptime of 99.9%, with individual uptimes for the intervals broken down according to the RR range given in Table 1.

**Table 1:** Performance for Different RR ranges. Performance at low (< 12), medium (12–20) and high (> 20) rates can be seen in the table

RR Interval (breaths/min)	RMSD (breaths/min)	Bias (breaths/min)	Uptime (%)	Number of RR Segments	Number of Samples
[4:11]	0.25	0.00	100	240	7155
[12:20]	0.76	-0.14	100	103	3075
[21:40]	0.96	-0.02	99.7	210	6195
**[4:40]**	**0.69**	**-0.03**	**99.9**	**553**	**16,425**

## Discussion

It is well known that respiratory rate is one of the least accurately recorded vital signs, with evidence suggesting that a common reason for this is because nursing staff believe that activities other than counting an accurate respiratory rate are more important for patient care. In a study of 79 registered nurses, Flenady et al. [18] found that, unless the patient was exhibiting signs of respiratory distress, was a pediatric patient, or had a history of respiratory related illness, other patient care activities were often prioritized over the accurate determination of respiratory rate. In fact, they found that quite often a chart would include an entry for respiratory rate without the rate having been assessed at all. This is a significant finding as abnormal respiratory rates are known to be an early indicator of life threatening physiological conditions and predictor of serious events such as cardiac arrest and admission to ICU [19]. Tracking accurate respiratory rate is therefore crucial in identifying early signs of deterioration.

According to Loughlin et al. [20] the manual measurement of respiratory rate in practice is conducted poorly because the importance of abnormal RR is not fully appreciated and a practice of estimating rather than measuring respiratory rate is prevalent. To solve this, the authors suggest the development of accurate, easy-to-use, inexpensive devices for the continuous monitoring of respiratory rate, and connected directly to the EHR. Elliott [21] echoes this sentiment citing the decision to measure RR depends on nurses’ perception of patient acuity, a lack of skill in assessing RR accurately and lack of automation and hence impact to workload as pertinent factors. Another contributing factor to the accurate manual assessment of RR is that patients may alter their breathing if they know they are being watched during manual assessment of RR (Kelly [22]) and surreptitious means of distracting their attention while doing so may be required. As with other authors, Kelly suggests the need for automation in this regard. The technology we are developing and reporting on here will address these issues and transform RR measurement from a poorly performed intermittent manual assessment to an accurate and continuous automated one. It fundamentally differs from other automated methods (e.g. capnography, spirometry, transthoracic impedance, acoustic, accelerometer, etc.), which require masks or canula at the face and mouth or sensors attached to the patient and thus do not lend themselves easily to a simple automated ubiquitous method for RR that does not interrupt clinician workflow.

In our study reported here, an RMSD accuracy of less than one breath-per-minute (0.69 breaths/min) was achieved with a corresponding near-zero bias for the continuous monitoring of respiratory rate. This high degree of agreement between the respiratory rate determined from the depth camera system and the reference devices corresponds well with results obtained from previous studies conducted by our group in healthy volunteers during a breathe-down study (used in pulse oximetry trials) [14] and subsequently in a longitudinal study of various breathing rates and patterns with a ventilator reference [15]. However, this present study covers a much wider range of respiratory rates and focuses on the accuracy at the low and high rates which are important in clinical practice. We are continually developing our prototype algorithm. The version used for this work was developed to cope with a wide range of respiratory rates while maintaining accuracy at the low and high rates. Previous prototype algorithms used in our earlier studies, were processed offline using manually identified regions of interest from which to extract the respiratory activity in the scene, whereas the current algorithm (1) is completely real-time in that it is now set up so that it can generate RR from the depth data streaming in and does not require post-processing offline, and (2) automatically finds the pertinent region of interest by locating the area within the scene where significant modulations occur and only targeting that region for the extraction of depth data from which to generate RR. We continue to work on the algorithm robustness including motion handling during gross patient movements in the scene.

**Fig. 4:**
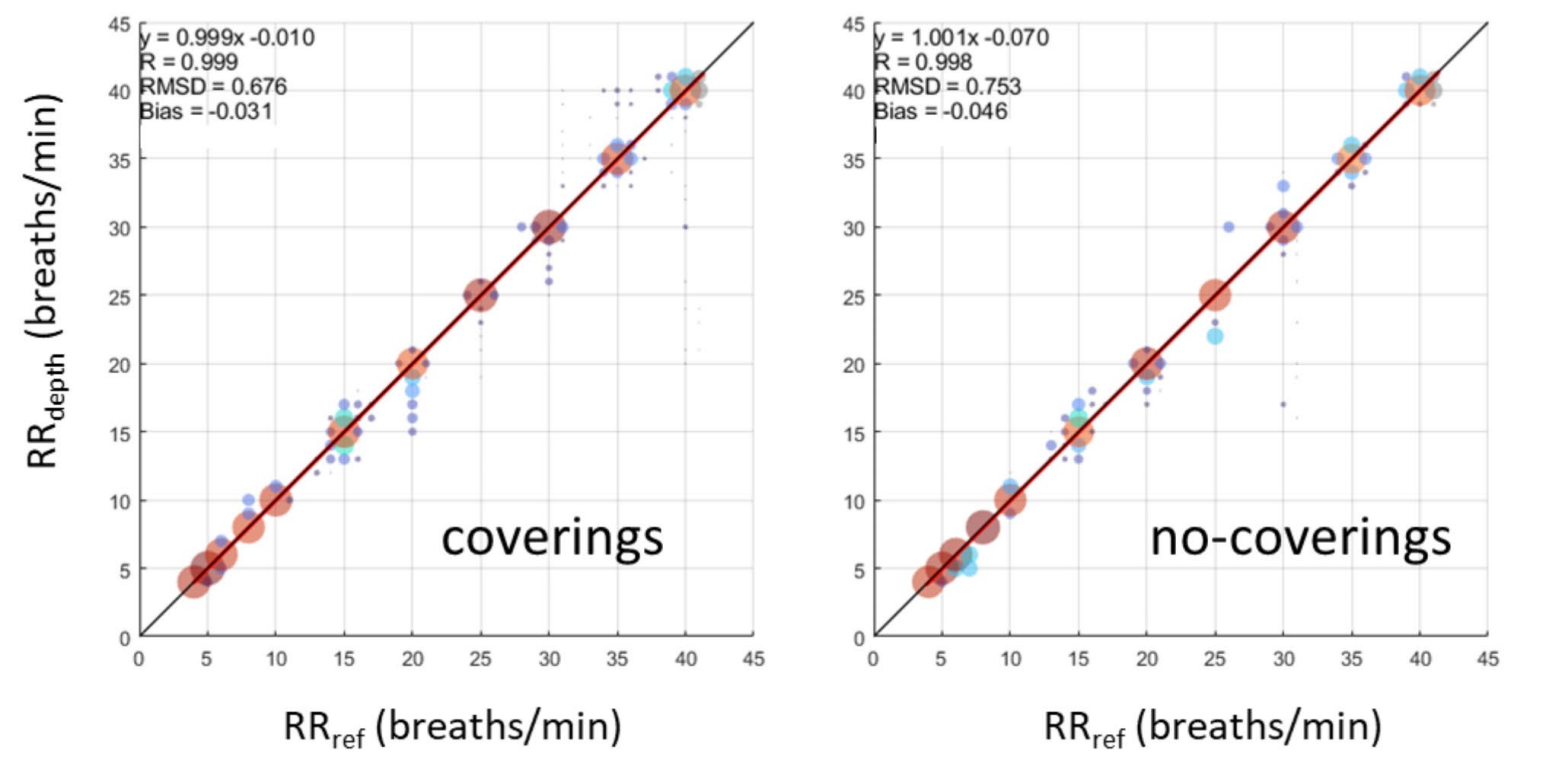
Results split according to coverings

It should be noted that we conducted the majority of our studies with the subjects under covers, as we noticed early on in algorithm development that this was a potentially more challenging condition. Thus 436 of the 553 respiratory rate segments used in the main analysis were conducted with covers. Figure 4 shows the scatter plots for the data split according to covering. It can be seen from the plot that there is effectively no difference in performance between the two sets with coverings and no-coverings producing low RMSD accuracies of 0.67 and 0.75 breaths/min respectively. Also, both sets lie almost exactly on the line of unity and exhibiting near unity correlation coefficients. This is obviously key for a clinically useful non-contact technology, as it must function when a patient is lying under bed covers.

There are a number of limitations to this study. A small number of participants (N = 7) took part, as the study was conducted during the COVID pandemic and access to more subjects was limited. As we progress the development of this technology, our aim is to test the system in cohorts of patients across a number of pertinent areas of care. These include: (1) patients with respiratory compromise who would be followed longitudinally over the course of their disease progression and their response to therapy; (2) patients in the recovery room recovering from anesthetic agents; (3) the sleep lab; and (4) patients in elderly care. However, a wide range of rates, bed coverings, room lighting conditions, postures, clothing, etc. were considered in order to include as wide a range of operating conditions as possible. The study did not investigate the effect of severe motion and/or caregiver interaction, this will be taken into account in future work. The study also had several strengths, for example: it only required an off-the-shelf depth camera with no hardware changes; the system requires no calibration and is simple to operate; high quality reference signals were obtained and used to calculate performance; and a wide range of clinically relevant rates were interrogated, resulting in a large dataset of over 550 separate rate measures with a corresponding accurate reference signal.

Other non-contact methods for measuring RR are available and our group have had experience using a number of them. Visible light-based methods perform poorly in low lighting and fail when no light is available. They also have challenges when respiratory motion is solely along the line of sight and/or if the color in the scene is homogenous and no moving element is clearly visible. Thermal methods - either considering the hot/cold cycle of exhaled/inhaled breath gas or at the skin around the nostrils - fail when there is no clear camera view of this effect, for example, when the patient is on his/her side, obscured by blankets or simply too small in the image to be seen clearly. Laser vibrometry requires a clear sight of the subject and accurate targeting. Radiofrequency sensors will pick up respiration but are much more sensitive to environmental noise including patient motions and other persons in range of the sensor (e.g. clinical staff working with and around the patient). A fuller account of these methods is provided by Massaroni et al. [23]. In contrast to many of these methods, we have found the depth system provides robust and accurate respiratory information across a wide range of rates and operating conditions, a necessary characteristic for the development of a clinically robust medical device.

Depth sensing respiratory rate has applicability across the clinical setting including the general care floor (GCF), post-anesthesia care unit (PACU), non-vented ICU patients, elderly care facilities, sleep facilities and at-home sleep studies. The technology also fits well with the current drive for more remote and non-contact monitoring of the patient stemming both from general industry trends and also the impetus from the recent global COVID pandemic [5, 6]. The incorporation of commercial camera systems within the hospital environment is becoming more prevalent. These are being deployed for a wide variety of tasks providing solutions, for example, for infection control (care.ai: https://www.care.ai/), heart rate and respiratory rate spot checks (Oxehealth https://www.oxehealth.com/) and bed exiting and fall prevention (Ocuvera https://ocuvera.com/). We believe that the deployment of a vision-based non-contact system for the robust and reliable monitoring or continuous RR is now possible.

## Conclusion

We believe that non-contact monitoring has great potential for the robust monitoring of a range of physiological and contextual parameters. The results reported here indicate the viability of continuous non-contact monitoring for the determination of one of these parameters - respiratory rate - over a clinically useful range of 4 to 40 breaths/min, where we have emphasized the performance at both clinically relevant low and high rates.
